# Structural Evidence of Active Site Adaptability towards Different Sized Substrates of Aromatic Amino Acid Aminotransferase from *Psychrobacter* Sp. B6

**DOI:** 10.3390/ma14123351

**Published:** 2021-06-17

**Authors:** Anna Bujacz, Jedrzej Rum, Maria Rutkiewicz, Agnieszka J. Pietrzyk-Brzezinska, Grzegorz Bujacz

**Affiliations:** 1 Institute of Molecular and Industrial Biotechnology, Lodz University of Technology, Stefanowskiego 4/10, 90-924 Lodz, Poland; jedrzej.rum@dokt.p.lodz.pl (J.R.); agnieszka.pietrzyk-brzezinska@p.lodz.pl (A.J.P.-B.); grzegorz.bujacz@p.lodz.pl (G.B.); 2 Macromolecular Structure and Interaction, Max Delbrück Center for Molecular Medicine, Robert-Rössle-Straße 10, 13125 Berlin, Germany; maria.rutkiewicz@mdc-berlin.de

**Keywords:** crystal structure, aromatic amino acids aminotransferase, active center adaptability, substrate specificity, psychrophilic, enzyme-inhibitor complexes

## Abstract

Aromatic amino acid aminotransferases present a special potential in the production of drugs and synthons, thanks to their ability to accommodate a wider range of substrates in their active site, in contrast to aliphatic amino acid aminotransferases. The mechanism of active site adjustment toward substrates of psychrophilic aromatic amino acid aminotransferase (*Psy*ArAT) from *Psychrobacter* sp. B6 is discussed based on crystal structures of complexes with four hydroxy-analogs of substrates: phenylalanine, tyrosine, tryptophan and aspartic acid. These competitive inhibitors are bound in the active center of *Psy*ArAT but do not undergo transamination reaction, which makes them an outstanding tool for examination of the enzyme catalytic center. The use of hydroxy-acids enabled insight into substrate binding by native *Psy*ArAT, without mutating the catalytic lysine and modifying cofactor interactions. Thus, the binding mode of substrates and the resulting analysis of the volume of the catalytic site is close to a native condition. Observation of these inhibitors’ binding allows for explanation of the enzyme’s adaptability to process various sizes of substrates and to gain knowledge about its potential biotechnological application. Depending on the character and size of the used inhibitors, the enzyme crystallized in different space groups and showed conformational changes of the active site upon ligand binding.

## 1. Introduction

Transamination stands as a bright example of reaction, which is nowadays conducted almost exclusively by biotechnological means. [1] Enzymatic methods allow to elude multistage and complicated chemical reactions and grant an enantiomerically pure product with high efficiency. Aromatic amino acid aminotransferases (ArAT) play a crucial role in the biosynthesis and degradation of various biomolecules. They participate in several essential metabolic pathways: methionine, tyrosine and phenylalanine metabolism; phenylalanine, tyrosine and tryptophan biosynthesis; novobiocin biosynthesis and alkaloid biosynthesis. [2] Although aminotransferases have been the subject of extensive research for more than half a century, their industrial importance has gradually grown from the early 2000s. The revolution started when the main problem of enzyme-driven transamination, which is unfavorable reaction kinetics, was overcome first by using substrate excess and then by product removal and by introduction of enzymatic cascade reactions. [3,4,5,6,7] Most of ArAT’s applications are related to ω-aminotransferases, as they are not limited to substrates with an amino group adjacent to the carboxyl one. However, the enantiomerically pure amino acids and their close derivatives are still in high demand. Thus, α-aminotransferases (αAts) remain industrially viable. [8,9,10,11,12] Interesting chemicals, which can be obtained via αAT-catalyzed reactions, are primary amines, such as L-tert-leucine, L-homoalanine, L-homophenylalanine, L-6-hydroxynorleucine, L-3-hydroxyadamantylglycine, L-2-aminooctanoic acid and L-2-naphtyl alanine. [13] Such a broad range of products is related to the high elasticity of Ats’ active pocket and/or improvements obtained by directed evolution of enzymes. [14,15] Another group of chemicals worth mentioning is the precursors for drugs, such as L-homoalanine, serving in the production of Ethambutol, Levetriacetam and Brivaracetam or amino acids and amines acting as pharmaceutics themselves, e.g., L-thienylalanine or L-norephedryne. Some of the enzyme-driven transamination products are useful in fields not related to pharmacy, for example, L-diphenylalanine, which is a substrate for the synthesis of peptide nanotubes that can be used as piezoelectric clean energy harvesters [16,17].

The awareness of the environmental impact of industrial processes, which is rapidly growing nowadays, puts psychrophilic enzymes in the spotlight. Cold-adapted enzymes show high activity, even at low temperatures, cutting the heat demand of the process. [18] Moreover, cold-adapted enzymes are a perfect solution for processes involving thermolabile substrates or products. Thus, structural analysis of such enzymes, based on crystal structures, is needed to fully understand the structure–function relationship and structural changes responsible for their function and potential usage. However, the search of the Protein Data Bank revealed a lack of such information for this group of proteins.

The psychrophilic aromatic aminotransferase from *Psychrobacter* sp. B6 is a representative of pyridoxal phosphate (PLP)-dependent enzymes. This vast group includes proteins belonging to five EC classes: oxidoreductases, transferases, hydrolases, lyases and isomerases. They are also subdivided into four fold-types; D-amino acid and branched-chain amino acid aminotransferases are members of the fold-type IV family; the rest of the aminotransferases, including aspartic and aromatic amino acid aminotransferases, such as that investigated in this work, *Psy*ArAT, are fold-type-I PLP-dependent enzymes. [19] To this day, investigated in this work, *Psy*ArAT is the only psychrophilic bacterial ArAT of known structure. [20] However, its mesophilic analog from *Paracoccus denitrificans* [21], thermophilic from *Pyrococcus horikoshii* [22] and fungal homolog from *Candida albicans* [23] were structurally studied.

*Psy*ArAT exhibits an activity typical for ArAT: transamination, which is a transfer of an amine group between aromatic α-amino acids and α-ketoacids. The reaction is described by a bi-bi ping-pong mechanism, due to its two half-stage reversible character. The first half of the reaction starts upon the binding of the catalytic lysine ε-amine group to the aldehyde group of PLP with the release of a water molecule and the creation of internal aldimine (PLI). The second step is an actual transamination, in which PLI is transformed into external aldimine (PLE) by the substitution of lysine with an amino acid. Next, PLE is hydrolyzed with the creation of α-ketoacid, corresponding to a substrate and pyridoxamine phosphate (PMP). In the second half-reaction, α-ketoacid reacts with PMP, giving an appropriate amino acid as a product and setting the cofactor back into the PLP state. This activity is of great importance for the metabolism of amino acids, enabling their degradation by conversion into citric acid cycle substrates and the synthesis of some nonessential amino acids [24].

**Figure 1:**
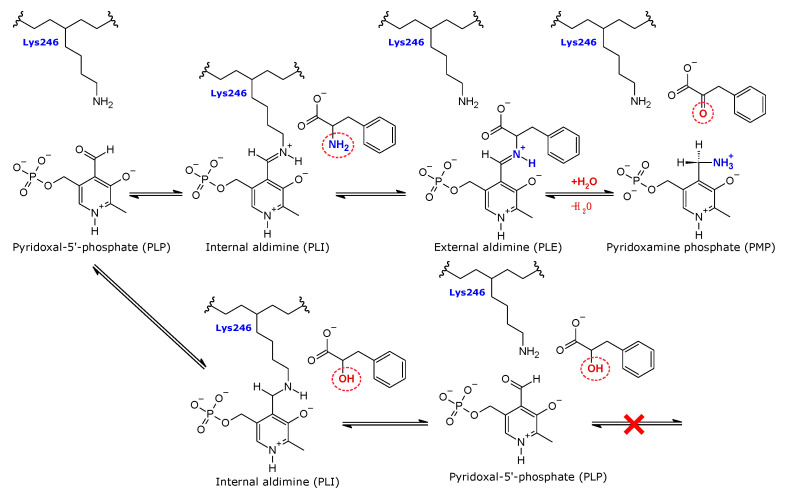
The mechanism of the transamination reaction, catalyzed by *Psy*ArAT (upper line), and broken by the presence of the inhibitor, hydroxy-analog of phenylalanine (lower line).

Hydroxy-analogs of amino acids, chosen for complex creation with *Psy*ArAT, are good candidates for the analysis of conformational changes occurring in the *Psy*ArAT active center during ligand binding. The only close bacterial homolog of *Psy*ArAT, of which structures in the form of complexes are available in the PDB (PDB IDs: 1AY5, 1AY8, 2AY2–2AY9) [25], is its mesophilic counterpart from *Paracoccus denitrificans*. Thus, all amino acid analogs used for bacterial aromatic aminotransferases’ structural and mechanistic proper-ties up to this point were carboxylic acids. We decided to use ligands having an amino group replaced by a hydroxyl group to keep the inhibitory character of an analog and enable its orientation to be presented in a manner mimicking the orientation of a real substrate, due to its ability to make an additional hydrogen bond with a Schiff base (Figure 1).

Due to the broad application of transamination in the chemical and pharmaceutical industry, it is important to provide comprehensive knowledge about the architecture and functions of transaminases obtained from extremophilic organisms, such as *Psychrobacter* sp. B6. Therefore, we found a way to show the active site of *Psy*ArAT in complexes, with its substrate structural analogs acting as inhibitors, to observe conformational changes in the catalytic pocket in the most similar surrounding as has place upon substrate binding. The four crystal structures of *Psy*ArAT complexed with hydroxy-analogs of enzyme substrates, i.e., phenylalanine (FOH), tyrosine (YOH), tryptophan (WOH) and aspartic acid (DOH), that are presented in this work, show great adaptability of its catalytic center towards binding various sized ligands. Moreover, a structural comparison of the investigated structures with native *Psy*ArAT (PDB ID: 4RKC) and with that complexed with aspartate, *Psy*ArAT/D (PDB ID: 4RKD), [20] provides an extended knowledge about interactions of the ligand within the catalytic center and the conformational changes of the enzyme active pocket.

## 2. Materials and Methods

### 2.1. Protein Preparation

The protein, used for obtaining *Psy*ArAT/FOH, *Psy*ArAT/YOH and *Psy*ArAT/DOH complexes, was prepared as described previously [24]. To improve the homogeneity of the protein sample and crystallization, a new expression construct, expression system and protein purification protocol was designed.

The *psyarat* gene was optimized for expression in *E. coli* (Eurofins, Luxembourg). It was amplified using Q5 High-Fidelity 2X Master Mix (New England Biolabs, Ipswich, MA, USA) and primers designed to be compatible with the pMCSG7 vector (AT_lic_fw, 5′-TACTTCCAATCCAATGCCATGTTCGAGCG-3′; AT_lic_rv, 5′-TTATCCACTTCCAATGTTAATCCTTGAGAACATC-3′). Cloning was performed using the ligation-independent cloning (LIC) method [26,27,28]; T4 DNA Polymerase, dGTP and dCTP were provided by New England Biolabs (Ipswich, MA, USA). DH5α competent cells were transformed with the resulting pMCSG7_*psyarat* vector using a basic heat shock procedure. Obtained transformants were cultivated overnight at 37 °C on LB-agar plates, with ampicillin as a selective marker. The plasmid was isolated using Promega kit and verified by sequencing (Genomed, Warsaw, Poland).

An inoculum of *ArcticExpress* (DE3) cells, transformed with pMCSG7_*psyarat* vectors, was cultivated with the addition of ampicillin (100 μg/mL) and gentamycin (20 μg/mL) overnight. TB medium was inoculated with 1% of the overnight culture, and protein overexpression was induced with 1 mM IPTG at OD ~0.6 and cultivated at 12 °C overnight. Pellet was collected by centrifugation at 3000× *g* at 4 °C for 20 min.

Cell pellets were resuspended in buffer A (20 mM TrisHCl, 500 mM NaCl, 20 mM imidazole, pH 7.4) supplemented with DTT at a final concentration of 100 mM. Cell lysis was performed by sonication using Bandelin Sonopuls GM 3200 (Bandelin electronic GmbH & Co. KG, Berlin, Germany). The extract was centrifuged for 60 min at 4 °C and 10,000× *g*. The clarified supernatant was then subjected to affinity chromatography using ÄKTA Pure system (GE Healthcare, Chicago, IL, United States) and HisTrap 5 mL column (GE Healthcare) equilibrated in buffer A. The tagged protein was eluted with buffer B (20 mM TrisHCl, 500 mM NaCl, 400 mM imidazole, pH 7.4). TEV protease digestion was performed overnight, parallel with buffer exchange to buffer A via dialysis. Reverse affinity chromatography was performed using the same setup to separate *Psy*ArAT from TEV protease and His_6_-tag. Size exclusion chromatography, with the usage of a HiLoad 16/600 Superdex 200 pg column (GE Healthcare) and buffer C (20 mM Tris-HCl pH 7.4, 100 mM NaCl), was performed to ensure sample purity necessary for crystallization. The *Psy*ArAT was concentrated to 12 mg/mL using Vivaspin20 (30.000 MWCO) concentrators (Sartorius, Göttingen, Germany). Each step of the purification was controlled by SDS-PAGE electrophoresis.

### 2.2. Crystallization

All crystallizations of the reported *Psy*ArAT complexes were performed by the hanging drop vapor-diffusion method at 291 K, using 24-well crystallization plates (Hampton Research, Aliso Viejo, CA, USA). Crystallization drops were set manually at 1 Μl + 1 μL, protein and well solution, respectively. The protein was concentrated to 9–11 mg/mL in 10 mM Tris–HCl buffer, pH 7.5, using Vivaspin concentrators with a 10 kDa cutoff (Sartorius, Göttingen, Germany). Hexagonal crystals of the *Psy*ArAT/DOH complex, with aspartic acid hydroxy-analog (DOH), were obtained in conditions consisting of 2.1 M DL-Malic acid, pH 7.0 and 0.1 M Tris-HCl buffer pH 7.5. Monoclinic crystals of *Psy*ArT/YOH and *Psy*ArAT/FOH were obtained by co-crystallization, using 0.2 M MgNO_3_, 20% PEG 2000, HEPES buffer, pH 7.5 and a 20-fold molar excess of L-tyrosine and L-phenylalanine hydroxy-analogs (YOH and FOH). C-centered monoclinic crystals of *Psy*ArAT/WOH were obtained by soaking native crystals, grown in the presence of 0.2 M MgNO_3_ and 20% PEG 3350 in HEPES buffer, pH 7.5, with a 5-fold molar excess of PLP and 10-fold molar excess of 3-indolelactic acid (hydroxy-analog of L-tryptophan–WOH), for 3 h.

### 2.3. Diffraction Data Collection, Structure Solution and Refinement

Diffraction measurements were carried out at 100 K temperature in the vapor stream of liquid nitrogen at HZB BESSY II synchrotron in Berlin, Germany. Crystals of *Psy*ArAT/DOH did not need any cryoprotectant, because they were obtained in the presence of dicarboxylic acid [29]. Diffraction data of *Psy*ArAT/DOH were processed in a hexa-gonal system to a resolution of 1.62 Å using HKL2000 [30]. The structure was solved in one of the enantiomorphic space groups, P6_5_22, using the monomer of *Psy*ArAT (PDB ID: 4RKC) as a model in Molrep [31]. Crystals of *Psy*ArAT/YOH, *Psy*ArAT/FOH and *Psy*ArAT/WOH were transferred for a moment to the drop containing a mixture of well solution and PEG 400 in a ratio of 1:1 before the diffraction experiment. For these complexes, diffraction data were processed using the XDSapp program. [32,33] *Psy*ArAT/YOH and *Psy*ArAT/FOH were indexed in monoclinic space group P2_1_ to a resolution of 2.31 Å and 2.50 Å, respectively, and *Psy*ArAT/WOH in C2 to a resolution 2.59 Å. The calculation of solvent content [34] showed that a monomer is present in the asymmetric unit of *Psy*ArAT/DOH, a dimer in the asymmetric unit of *Psy*ArAT/YOH and *Psy*ArAT/FOH and two dimers in the case of *Psy*ArAT/WOH. The structures of *Psy*ArAT/YOH and *Psy*ArAT/FOH complexes were solved by molecular replacement in Phaser [35], using the dimer of the native enzyme as a model. The structure of *Psy*ArAT/WOH was solved by molecular replacement in Molrep [31] using the dimer of *Psy*ArAT (PDB ID: 4RKC) as a model. For all crystal structures, the refinement was performed in REFMAC5 [36] from the CCP4I suite [37] with TLS restrains [38]. Structures were rebuilt using graphical program COOT [39] and validated using MolProbity programs [40,41]. Statistics details of the diffraction experiment, diffraction data reduction and crystal structure refinement are presented in Table 1.

## 3. Results and Discussion

### 3.1. Overall Structure of PsyArAT Complexes

**Figure 2:**
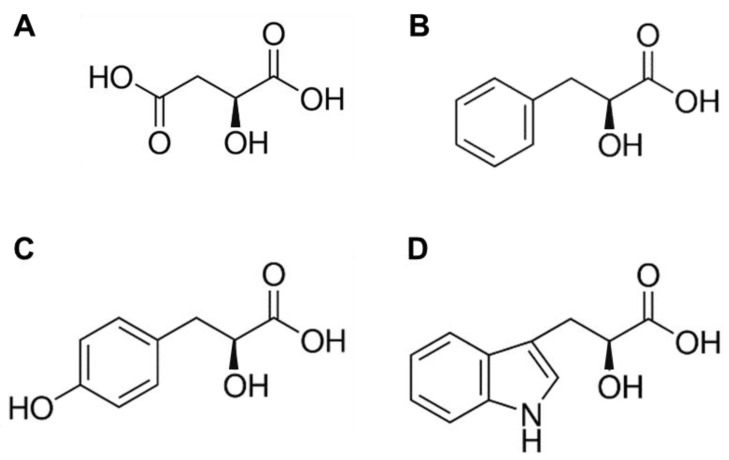
Hydroxy-analogs of *Psy*ArAT substrates: (**A**) L-malic acid (DOH, MLT); (**B**) L-3-Phenyllactic acid (FOH, HFA); (**C**) L-p-Hydroxyphenyllactic acid (YOH, TYF); (**D**) L-3-indolelactic acid (WOH; 3IL).

Four crystal structures of *Psy*ArAT in complexes with substrate hydroxy-analogs, L-malic acid (DOH), 3-phenyllactic acid (FOH), 3-(4-hydroxyphenyl)-lactic acid (YOH) and 3-indolelactic acid (WOH) (Figure 2), were determined to 1.62 Å, 2.31 Å, 2.52 Å and 2.59 Å resolution, respectively. The aforementioned complexes crystallized in three space groups: hexagonal P6_5_22 for *Psy*ArAT/DOH, monoclinic P2_1_ for *Psy*ArAT/FOH and *Psy*ArAT/YOH and C2 for *Psy*ArAT/WOH, as a result of different crystallization conditions and the presence of ligands varying in size and chemical character.

**Figure 3:**
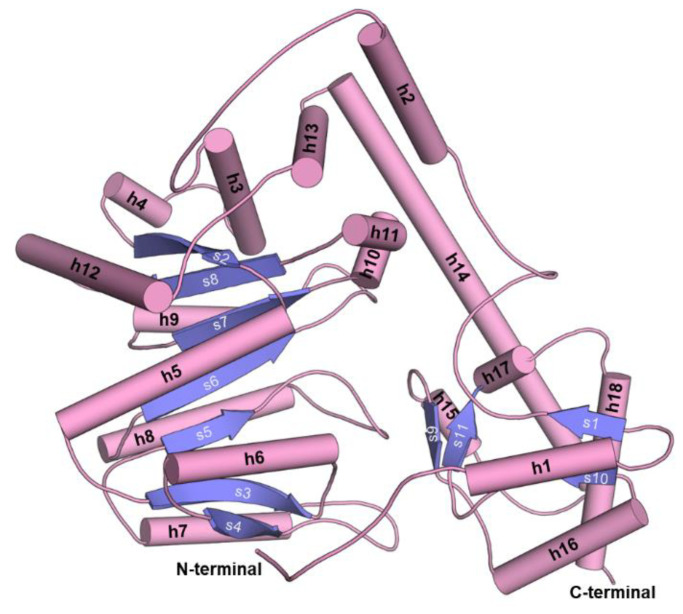
Secondary structure elements in *Psy*ArAT monomer.

The overall architecture of the *Psy*ArAT is typical for the type-I PLP-dependent enzymes and was previously described [24]. The dimer, functional unit of the enzyme, has an ovaloid shape with a 2-fold noncrystallographical symmetry axis (in the case of *Psy*ArAT/DOH, it is also a crystallographic axis) laying along with its height (~45 Å) and dividing the homodimeric structure into two identical subunits. The longest dimension of ~100 Å crosses over both monomers. Subunits are connected by salt bridges created between the carboxylic group of Glu253 and α-amino nitrogen of His289, as well as by side chains of Arg63 and Glu53 and by hydrophobic interactions between Ile101 of adjacent monomers and anchoring residues of the N-terminal coil (Met1, Phe2 and Ile5). The interface area between monomers measured 3112 Å^2^. Each subunit is divided into two domains differing in function and architecture. A flexible small domain, responsible for closing of the active site during ligand binding, is built of both N- and C-termini of the protein, involving residues Met1-Leu66 and Pro286-Asp398. Both parts are connected by short parallel β-sheets: β-strand1 (β1) Val29–Leu31 and β-strand10 (β10) Gly366–Tyr368, which allow for the simultaneous movement of the mentioned protein N- and C-terminal fragments (Figure 3). The large domain is a conformationally stable part of the monomer; its architecture can be described as a Rossmann-like α-β-α sandwich fold, which is related to the main function: PLP binding and stabilization. This kind of arrangement is typical for nucleotide-binding proteins, and *Psy*ArAT’s interaction with pyridoxal phosphate could be explained as analogous to interactions at the active sites of FAD and NAD(P) binding proteins, e.g., phosphoglycerate dehydrogenase or glutathione reductase, where ligands are also bound by a phosphate group and nitrogen of a heterocyclic aromatic ring [42].

**Figure 4:**
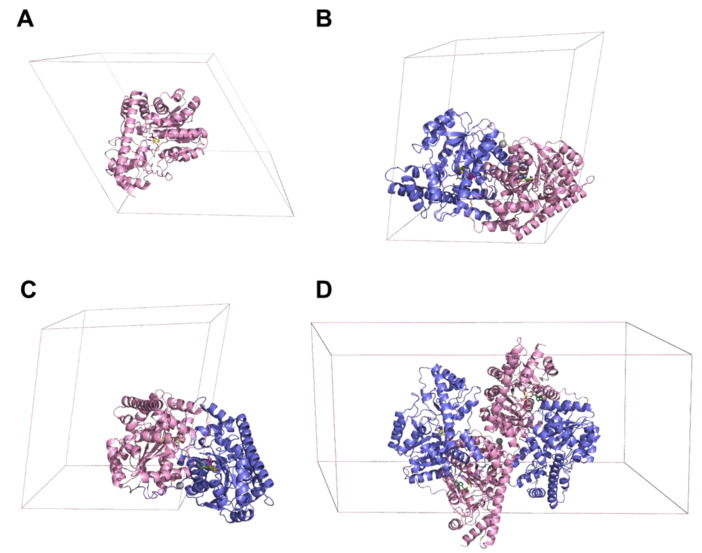
Unit cells and asymmetric units (ASU) of *Psy*ArAT complexes. (**A**) *Psy*ArAT/DOH (P6_5_22), monomer; (**B**) *Psy*ArAT/FOH (P2_1_), dimer; (**C**) *Psy*ArAT/YOH (P2_1_), dimer and (**D**) *Psy*ArAT/WOH (C2), two dimers.

Depending on the crystal form of *Psy*ArAT, the determined complexes have different asymmetric units (ASU). In the case of *Psy*ArAT/FOH and *Psy*ArAT/YOH, the ASU is created by a functional dimer; in *Psy*ArAT/DOH the asymmetric unit represented only a monomer, but the functional dimer is reproduced with the symmetry-related molecule. The two-fold axis of the dimer is collinear with the crystallographic diagonal two-fold axis. In the *Psy*ArAT/WOH complex, two functional dimers are present in the asymmetric unit (Figure 4).

### 3.2. The Active Site of PsyArAT Complexes with Hydroxy-Analogs of Substrates

Entrances to the active pockets create two grooves on the opposite sites of the protein surface. Each monomer contains an equivalent active center with pyridoxal-5′-phosphate (PLP); however, depending on the reaction stage, the cofactor may have taken a different form. In *Psy*ArAT/FOH, *Psy*ArAT/YOH and *Psy*ArAT/DOH complexes, fractional occupancy for PLP was observed. In *Psy*ArAT/WOH, PLP was added to the crystallization solution, which helped to obtain very good electron density maps for the cofactor.

The PLP molecule is bound in the enzyme active center by an extensive network of hydrogen bonds. The only nonpolar interaction holding PLP is π-stacking of the pyrimidine ring with the indole ring of Trp130. Conformation of the amine group of Lys246, which is essential for transamination catalysis, depending on the stage of the reaction, can create a hydrogen bond to an entity present in position C4 of the cofactor, the formyl group of PLP, the amine group of pyridoxamine phosphate (PMP), or a covalent bond with internal aldimine (PLI) [24].

While the creation of the internal aldimine is not caused by direct contact with a substrate (or inhibitor), as this is an initial step enabling their eventual further conjugation, it seemed to be triggered by the presence of the ligand in the pocket. Interestingly, although all hydroxy-analogs of substrates used for the creation of *Psy*ArAT/FOH, *Psy*ArAT/YOH, *Psy*ArAT/WOH and *Psy*ArAT/DOH complexes acted as competitive inhibitors, the active sites should be essentially at the same pre-reaction stage; however, the form of the cofactor was not uniform among the pockets. The internal aldimine (PLI) (covalently bound PLP to Lys246) was observed in the *Psy*ArAT/DOH, in all of the four pockets of *Psy*ArAT/WOH and the one monomer of *Psy*ArAT/YOH and *Psy*ArAT/FOH homodimers. Despite the presence of an inhibitor in the pockets of chain B of *Psy*ArAT/YOH and *Psy*ArAT/FOH dimers, the non-bound form of PLP was present, similarly, as it was in the catalytic center at both monomers of the native enzyme structure. In the *Psy*ArAT/FOH complex, the closest distance between the ligand and PLP (measured from the α-hydroxyl group of the inhibitor to aldehyde carbon of PLP) was 4.1 Å in monomer A and 3.3 Å in monomer B, while in the *Psy*ArAT/YOH structure respective distances were bigger (5.7 Å and 4.6 Å). The pockets in monomers A of these two complexes were also larger in volume than the centers of chains B, the center of *Psy*ArAT/DOH and centers of *Psy*ArAT/WOH monomers with bound WOH. Thus, it can be concluded that strong binding of the ligand leads to a more compact structure, with the cofactor formed in a pre-reactionary stage.

### 3.3. PsyArAT Interactions with Hydroxy-Analogs of Substrates

**Figure 5:**
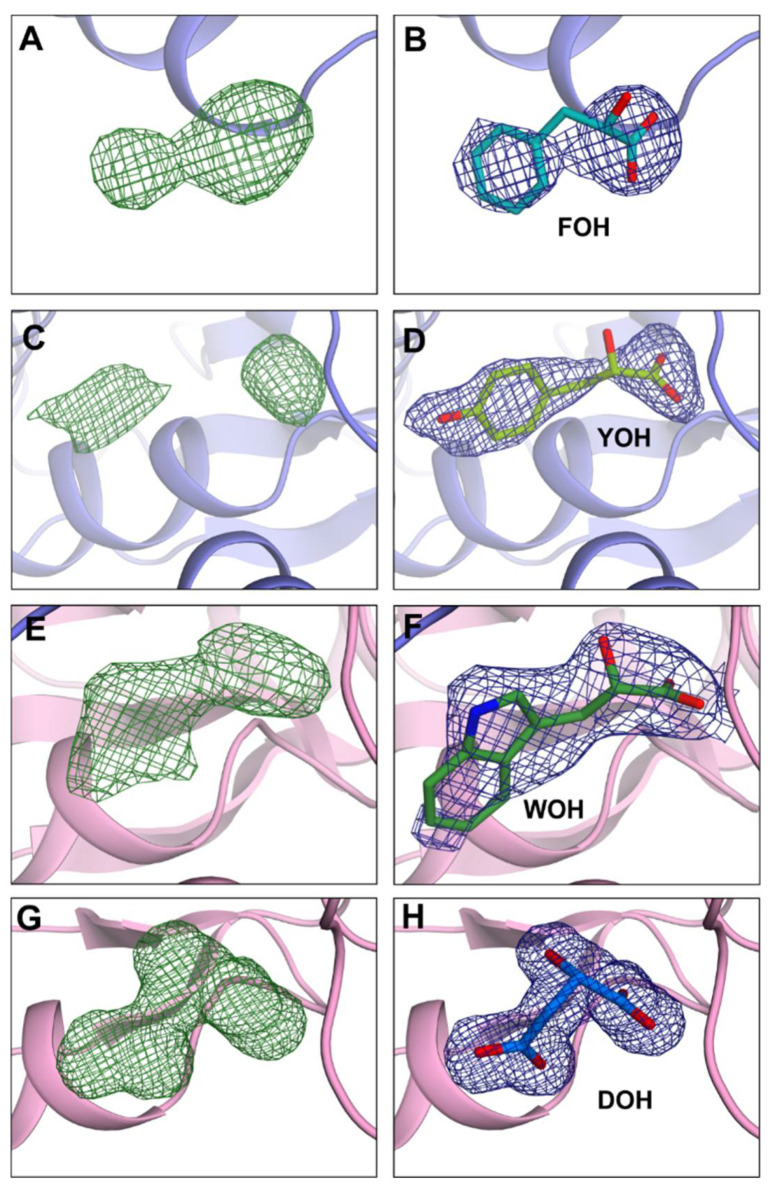
Unit cells. The electron density maps for ligands in *Psy*ArAT complexes. (**A**,**C**,**E**,**G**) The electron density omit map *Fo-Fc*, indicating the presence of FOH, YOH, WOH and DOH, respectively. (**B**,**D**,**F**,**H**) The electron density map *2Fo*-*Fc* displayed on FOH, YOH, WOH and DOH, respectively. The electron density maps *Fo-Fc* (green) were contoured at 3.0 σ, and *2Fo-Fc* were displayed in blue at 1.0 σ.

The hydroxy-analogs of *Psy*ArAT substrates used in this work (Figure 1) were clearly visible in electron density maps (Figure 5) of the solved structures. Ligands interact in the enzyme active center mostly by hydrogen bonds between their carboxylic groups, with the guanidine group of Arg374 belonging to the C-terminus and the amine group hydrogen of Gly34 belonging to the N-terminus of the small domain. They also created polar and hydrophobic contacts with two residues of the large domain: Asn183 and Trp130. Such a set of amino acids involved in inhibitor binding allows for the formation of the closed protein conformation by bringing together both the large and the small domains.

**Figure 6:**
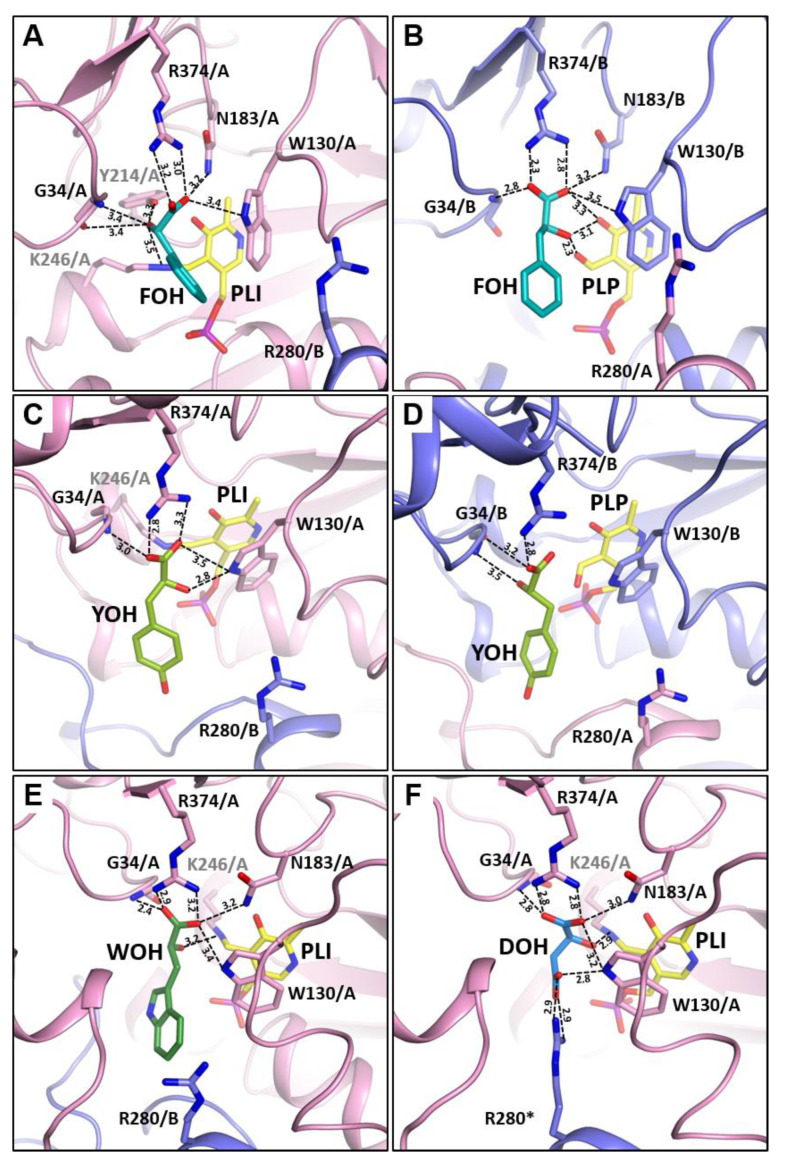
Interactions of ligands in the active center of *Psy*ArAT complexes: (**A**,**B**) *Psy*ArAT/FOH in monomer A and B, respectively; (**C**,**D**) *Psy*ArAT/YOH in monomers A and B, respectively; (**E**) *Psy*ArAT/DOH in monomer A; (**F**) *Psy*ArAT/WOH in monomer A. (Chain A is shown in pink and B in blue in all presented structures.)

The hydrogen bonds network made by a FOH ligand were slightly different for both monomers of the *Psy*ArAT/FOH complex (Figure 6A,B). In either case, carboxyl group of FOH creates two parallel hydrogen bonds with the guanidine group of Arg374 (3.2 Å and 3.0 Å in monomer A, 2.3 Å and 2.8 Å in monomer B), one with a side-chain nitrogen of Asn183 (3.2 Å for both monomers) and one with an indole ring nitrogen of Trp130 (3.4 Å for both pockets). Interactions between the ligand carboxyl group and the cofactor was present only in pocket B, where it had contact with formyl oxygen (3.1 Å). The interaction with the peptide bond nitrogen of Gly34 was made by the carboxyl oxygen of FOH (2.8 Å) in the active site of pocket B, while the ligand in the neighboring subunit made contact with the same residue, using its hydroxyl group (3.4 Å). Generally, most of the differences were a result of different placement of the -OH group in the pockets due to a rotation of ligand by 90°. Thus, in monomer A, besides the aforementioned interaction with Gly34, the hydroxyl group of FOH made bonds with the carbonyl oxygen of Gly34 (3.4 Å), the hydroxyl oxygen of Tyr214 (3.3 Å) and PLI Schiff base nitrogen (3.5 Å). In pocket B, the -OH of the ligand made the second contact with Trp130 (3.3 Å) and created two hydrogen bonds with a PLP molecule, first with the aldehyde group (2.3 Å) and the second with the hydroxyl oxygen of the cofactor (3.1 Å).

Despite the high chemical similarity to FOH, the interaction of the YOH inhibitor with the residues forming the active site of subunit A is slightly weaker, and only five hydrogen bonds were created, two with the guanidine moiety of Arg374 (2.8 Å and 3.3 Å), one with the peptide nitrogen of Gly34 (3.0 Å) and another two with the indole nitrogen of Trp130 (the first like all of those mentioned, by a carboxyl group (3.5 Å), and the second by a hydroxyl group of YOH (2.8 Å)) (Figure 6C). In the pocket B, the ligand made a weaker network of hydrogen bonds, interacting only with one nitrogen atom of the Arg374 guanidine group (2.8 Å) and the carbonyl oxygen of Gly34 (3.2 Å), by its carboxyl group and by its hydroxyl group with Gly34 nitrogen (3.5 Å) (Figure 6D). In the catalytic center of both complexes, the Arg280* switch was in the “down” position; however, in the case of *Psy*ArAT/YOH, it was placed a little deeper than in the *Psy*ArAT/FOH complex. In the subunit A, Arg280*, which was pushed down by a YOH ligand, made two hydrogen bonds between guanidine moiety nitrogen atoms and carboxyl oxygens of Asp11 (2.8 Å and 3.5 Å). In monomer B, only one contact was present with Asp11 (2.9 Å), but another bond was created with the hydroxyl group of Asn132 (3.5 Å). As stated, in *Psy*ArAT/FOH complex, Arg280* was placed slightly further from the ligand; in the active site of monomer A, it interacted with Asp11 (2.8 Å) like in the previous case, but it also reached Tyr8 hydroxyl oxygen (3.2 Å). In the active pocket of monomer B, Arg280* made an even shorter bond with the OH group of Tyr8 (2.8 Å), but the interaction with Asn132 (2.9 Å) replaced the one with Asp11.

Tryptophan hydroxy-analog (WOH) created a network of contacts, enabling the involvement of both termini and a large domain, which resulted in closure conformation. High similarity of interactions with an inhibitor in catalytic centers of monomers A and C allowed for the description of only one of them, and we arbitrary choose monomer A. The carboxylic group of the WOH is planar to the Arg374 guanidine group, and two hydrogen bonds of 3.2 Å and 2.9 Å are made between them. The another contact in the length of 2.4 Å, of the mentioned carboxyl group, was made with the peptide bond hydrogen of Gly34, being part of the motile, long entrance loop. Additionally, side chain nitrogen atoms of Trp130 and Asn183, from the large domain, make hydrogen bonds (3.4 Å and 3.2 Å) with the carboxyl group of the ligand. Internal aldimine present in the catalytic center interacts with the carboxyl group of the WOH; the bond between the Schiff base nitrogen and oxygen atom is 3.2 Å long (Figure 6E). Like in the case of the remaining complexes with aromatic ligands, FOH and YOH, Arg280*, from the second monomer, is in the “down” position, and one of its terminal nitrogen atoms creates two hydrogen bonds with side chains of Tyr8 and Asp11 (3.2 Å and 2.6 Å).

DL-malic acid sodium salt was used for the *Psy*ArAT/DOH complex creation, but only L-malic acid (DOH) was bound in the active center. The ligand creates considerably more hydrogen bonds than aromatic inhibitors; 10 interactions with residues in the active site were present (Figure 6E). Oxygen atoms of the α-carboxylic group of malic acid create strong parallel hydrogen bonds with two nitrogen atoms of the guanidine group from Arg374: (2.8 Å and 2.8 Å). Additionally, they are involved in contact with the amide group of Gly34 (2.8 Å) and Asn183 (3.0 Å). Oxygens of the β-carboxylic group of the ligand interacted, via hydrogen bonds, with Arg280* (2.9 Å and 2.9 Å) and Trp130 (2.8 Å). Hydroxyl oxygen of malic acid creates weak hydrogen bonds with Trp130 (3.2 Å) and stronger bonds with Nζ of Lys246 (2.9 Å) (Figure 6F). Interaction with Arg280* was of special importance; in this situation, the arginine switch was in the “up” position, and the ligand is shoved further from the entrance; hence, the helix H1 is able to close the active site fully. In this position Arg280* is still interacting with Asp11 (2.8Å, 3.4 Å) from the N-termini domain.

### 3.4. Arginine Switch

**Figure 7:**
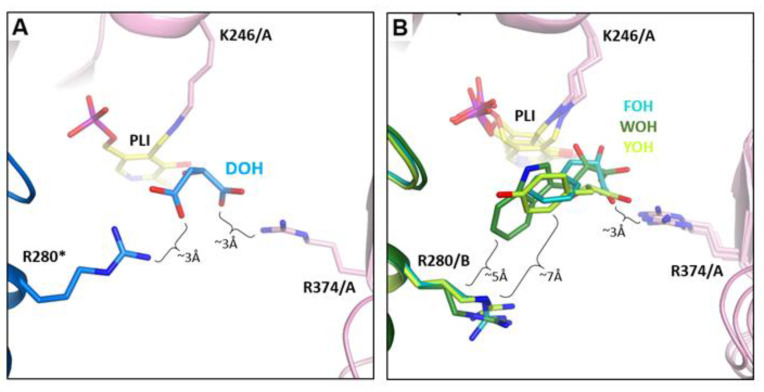
Arginine (Arg280*) “switch”; (**A**) in “up” position in *Psy*ArAT/DOH and (**B**) in “down” position in *Psy*ArAT/FOH, *Psy*ArAT/YOH and *Psy*ArAT/WOH complexes.

The action of Arg280* of the adjacent monomer (*) is important for the control over volume and substrate specificity of the *Psy*ArAT active pocket [43]. The long electrophilic side chain of this “arginine switch” can take one of two positions, “up” or “down”, due to a guarding interaction with Asp11 and Asn132, responsible for limiting its conformational freedom. In the “down” position, additional contact with Tyr8 can be made. This hydrogen bond system allows for both control of the chemical environment and the volume of the active site. When an aromatic hydrophobic inhibitor or a substrate was present in the active center, Arg280* took the “down” position, allowing for the entrance of the hydrophobic ligand and creates a sterical hindrance between helices H1 and H6, preventing the closing of the side entrance and making the pocket more spacious (Figure 7). The “up” position was taken when dicarboxylic ligand, such as malic acid, entered the active pocket. A lack of sterical hindrance enabled for the full closure conformation, and additional interactions with the ligand were made due to the presence of the guanidine group of Arg280 in the vicinity of the active site (Figure 7).

Interestingly, in the active site of monomer A and C in the *Psy*ArAT/WOH complex, the presence of a big, hydrophobic indole group of the WOH molecule, just near the Arg280* residue, prevented its contact with the Asn132 side chain (which was pushed away from the active pocket). Seemingly, such interaction was not necessary, as the conformational freedom of Arg280* was limited by the inhibitor itself. However, the carboxyl group of Asp11 interacted with the guanidino group of Arg280* via two (2.8 Å and 3.4 Å) hydrogen bonds.

### 3.5. Active Pocket Volume and Area of the PsyArAT in Native and Complexes Form

Contrary to all structures of aromatic aminotransferases complexed with inhibitors which are currently published, conformational changes in *Psy*ArAT monomers seem to be induced exclusively by a ligand presence. Unit cell dimensions for the native enzyme *Psy*ArAT, *Psy*ArAT/FOH and *Psy*ArAT/YOH complexes are nearly the same, despite motions occurring in domains. Moreover, calculations done using PISA [44] showed differences in the areas of contact made by each subunit in dimeric structures. For the *Psy*ArAT/YOH complex, the difference in the interface area between subunits A and B is only 14.5 Å^2^, in favor of domain B, while for *Psy*ArAT/FOH, it was 87.2 Å^2^, with more contacts for monomer B as well. However, the conformational changes for these two complexes did not seem to be in relation to these values, as the *Psy*ArAT/YOH complex showed bigger differences between monomers. Moreover, putting monomers B of these three structures in order from the most closed to the most open, which was *Psy*ArAT/FOH, *Psy*ArATYOH and the native enzyme, did not correlate to the order of contact area from lowest to the highest: native, *Psy*ArAT/FOH and *Psy*ArAT/YOH. The *Psy*ArAT/DOH complex could not be so easily compared to the structures with analogs of phenylalanine and tyrosine, as it crystallized as a monomer in ASU in a hexagonal unit cell; however, the areas of contact (excluding the one being the dimer interface) was about 720Å, which was similar to values for *Psy*ArAT/FOH, being much more relaxed structurally. *Psy*ArAT/WOH was another complex with different crystallographic properties, as it crystallized in a C2 group as a double dimer with noncrystallographical symmetry. In the case of this complex, it is possible that crystal packing affected the conformation, as a difference in the area of contact with neighboring proteins in a lattice was high (~500 Å^2^), where more contacts were made by closed domains.

**Figure 8:**
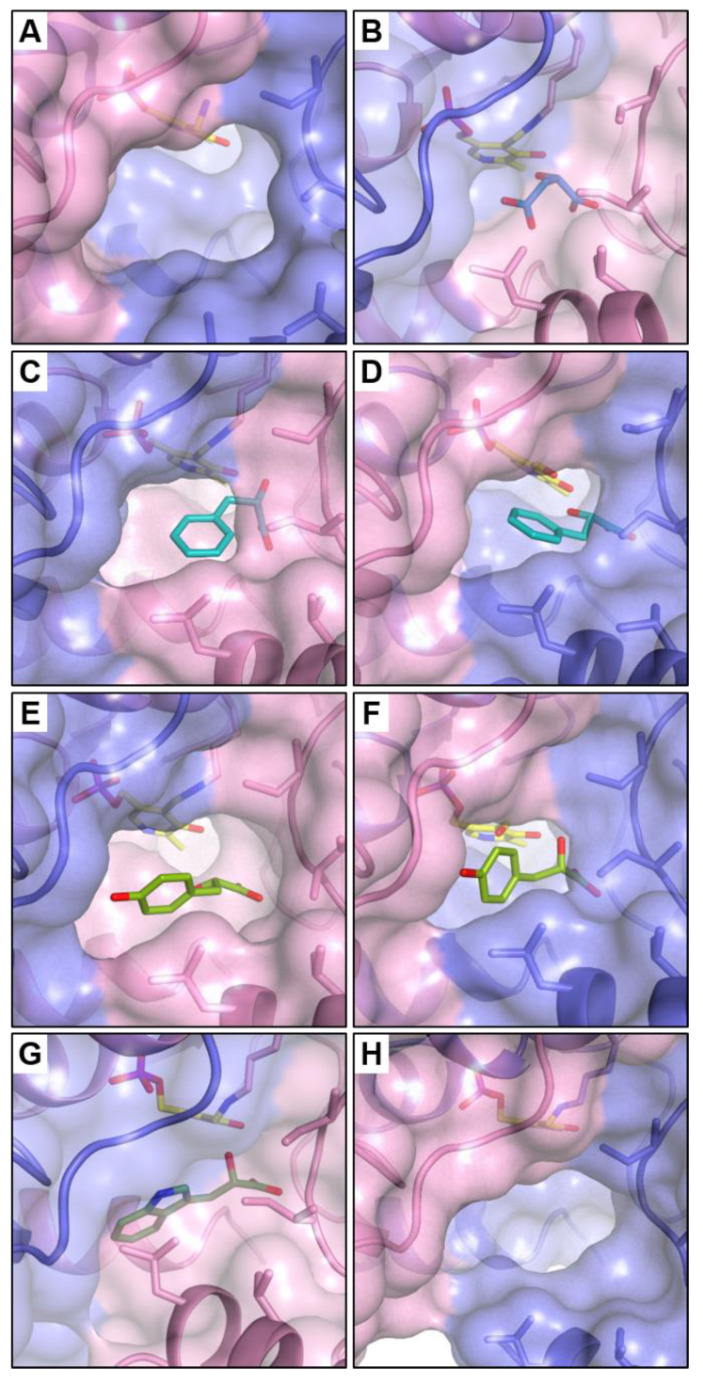
Entrances to the active pockets of (**A**) native *Psy*ArAT; (**B**) *Psy*ArAT/DOH; (**C**,**D**) *Psy*ArAT/FOH in A and B monomers; (**E**,**F**) *Psy*ArAT/YOH in A and B monomers; (**G**,**H**) *Psy*ArAT/WOH in A and B monomers.

The different position, interactions and presence or lack of the ligand in the active center of the studied complexes prompted us to investigate the volume and area of the active pockets and entrances to it in all monomers. Volumes were measured using the CASTp 3.0 tool [45], with probe size set to 1.2 Å. Interestingly, the volume of the pockets in one monomer was much smaller than in the second one of the dimer in all complexes (excluding *Psy*ArAT/DOH), but the difference in the case of FOH bound caused a smaller disparity between them than in the case of YOH present in the active center (Table 2) (Figure 8C–F). The *Psy*ArAT/DOH active site is considerably smaller (Figure 8B, Figure S1) in comparison to the two mentioned. Baffling was a lack of WOH in one monomer of each dimer in *Psy*ArAT/WOH, which resulted in a huge difference in the volume of the active pocket, with the tryptophan hydroxy-analog present or absent in the active center (Figure 8G,H). Even more surprising were the twice bigger values of the volume and surface of the active pocket lacking an inhibitor in comparison to the one occupied by the ligand. Analyzing these parameters with the native enzyme (Figure 8A), we found that the fully opened pocket of the native *Psy*ArAT was about two times bigger in comparison to pockets in *Psy*ArAT/FOH and PsyArAT/YOH complexes, two times bigger than for *Psy*ArAT/WOH and four times bigger than for *Psy*ArAT/DOH (Table 2).

Hence, it can be concluded that the volume of the *Psy*ArAT active center differs depending on the conformational states adopted by the enzyme, and on the size of the ligand bound in the active pocket. These open, closed and semi-closed conformations were also related to structural motions triggered by the presence of a specific ligand.

### 3.6. PsyArAT Motions upon the Binding of the Substrate Hydroxy-Analogs

**Figure 9:**
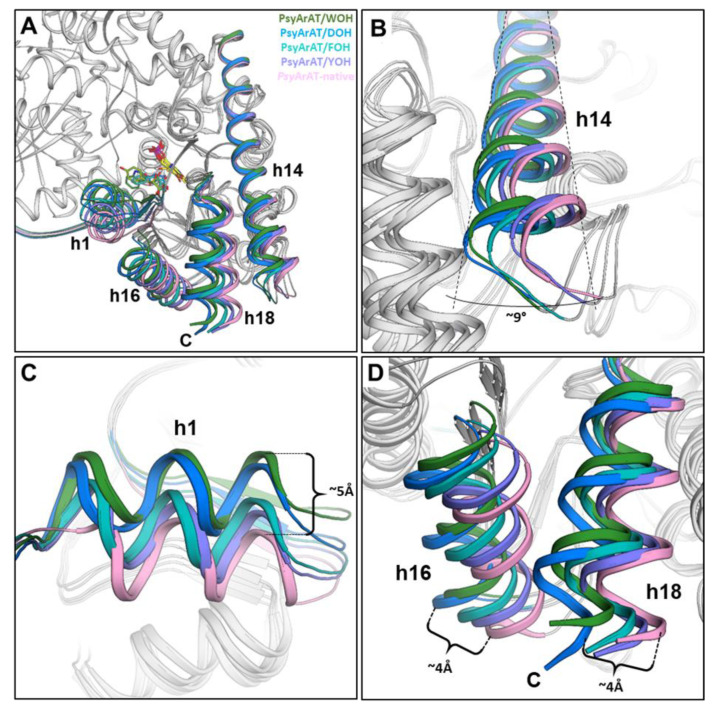
*Psy*ArAT domains’ motion during ligand binding. Overall view of the aligned structures (**A**); Kink of the long helix h14 (**B**); the motion of the helix h1 (**C**); Synchronic movement of the helix h16 and h18 (**D**). Values of domain movement are in Table 2.

The binding of different types of ligands caused conformational adjustment of the *Psy*ArAT monomers/domains in relation to each other. Generally, during the pocket closing, three main motions occurred in the enzyme structure: kink on a long helix H14 and two perpendicular to each other and shear motions of the small domain main components: N-terminal H1 helix and C-terminal helices (H16, H17 and H18) towards the pocket entrance (Figure 9). Using the open form of monomer B of the native *Psy*ArAT as a reference, approximate lengths of these movements were measured. The biggest differences in respect to the structure with no ligands bound (except for the cofactor) were recognized for *Psy*ArAT/DOH and *Psy*ArAT/WOH. Namely, the kink on the helix H14 was estimated at 9 and 8.5 degrees for two complexes, respectively, measured motions of small domain C-terminal helices were equal 3.8 and 3.3 Å and for N-terminal helix H1, these values equated to 4.5 and 5.2 Å in the aforementioned complexes. Despite the similar size and aromatic character of FOH and YOH ligands, motions in the structure of *Psy*ArAT complexed with the phenylalanine analog were slightly more significant. The kink on the long helix H14 was at least two times bigger for *Psy*ArAT/FOH (~6° in monomer A and ~4.5° in monomer B) than for *Psy*ArAT/YOH (~1.5° in monomer A and ~2.0° in monomer B). Movements of the N-terminal (~2.7 Å/A, ~3.0 Å/B) and C-terminal (~2.2 Å/A, 1.7 Å/B) parts of the small domain were also bigger for the *Psy*ArAT/FOH complex than for *Psy*ArAT/YOH (~1.2 Å/A, 1.8 Å/B and ~0.5 Å/A, ~1.0 Å/B, respectively).

The shear motion of the helix H1 is crucial for the closing of the pocket, due to the presence of hydrophobic Ile13, Leu14 and Val17 residues being part of it. During movement of the helix H1, simultaneous rotation took place, which caused a shift of the amino acids towards the entrance from the outer region; thus, an impervious water plug was created. The side chain of Ile33 also helped to plug the entrance because of the Asn25–Val45 long loop movement, which is coordinated with the small domain C-terminus shear motion. Leu14 is responsible not only for providing the hydrophobic environment, but also acted as a “probe”, sensing the size of a bound ligand. In complexes of *Psy*ArAT/FOH, *Psy*ArAT/YOH and *Psy*ArAT/WOH, the leucine side chain was unable to move further into the enzyme catalytic pocket due to a sterical hindrance made with the inhibitor molecule. DOH is a ligand of smaller size than aromatic hydroxy-analogs, and due to interactions with Arg280*, it was shoved deeper in the active site; in this situation, Leu14 moved into the center, enabling proper rotation of H1 and full closure conformation (Figure 9).

In the previously determined structure of *Psy*ArAT in the complex with aspartic acid (PDB ID: 4RKD), four dimers were present in the asymmetric unit. This structure represented a different stage of transamination reaction; the conformation of the monomers in each dimer was not identical, but more similar than in complexes studied in this work (RMSD values for Cα in a range 0.40–0.49Å). This indicated that processing of the small size substrate does not require a big conformational movement of the whole functional unit. A similar observation was noticed for two structures of complexes with ligands of medium size (FOH and YOH), where the ligand was present in the active center of both monomers (RMSD for Cα of 0.46 Å and 0.42 Å, respectively). In the *apo* structure of *Psy*ArAT (PDB ID: 4RKC), the conformational changes of monomers were larger (RMSD for Cα of 0.79Å). These differences indicates that one of the active sites was more open to accommodate the potential substrate. More significant conformational differences of monomers in dimers of *Psy*ArAT/WOH are visible, where the RMSD for Cα between monomers are around 1.07 Å and 1.30 Å (Table 1). This observation was made in a number of other structures of the PLP-dependent enzyme, which clearly indicates domain motion depending on the reaction stage.

**Figure 10:**
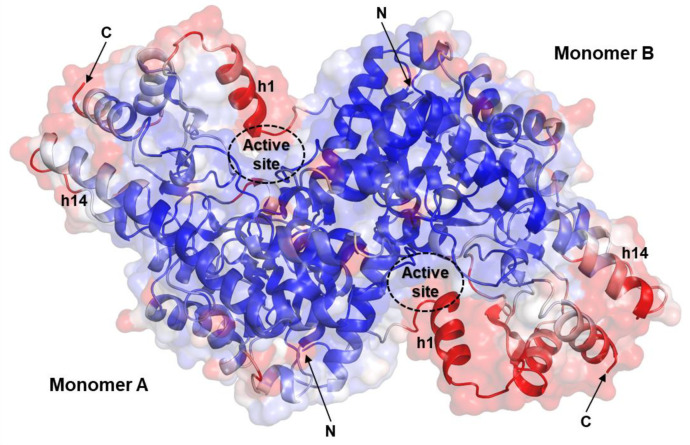
Atomic displacement parameters of *Psy*ArAT dimer (PDB ID: 4RKC); the low B-factors are colored in navy blue, and the high B-factors are colored in red.

Most of atoms, especially in the enzyme center of *Psy*ArAT, presented a low value of the displacement parameter, but chains attending in ligand incorporation to the active pocket have considerably higher average B-factors, which indicates their motility during transamination reaction (Figure 10).

### 3.7. Comparing Structures of PsyArAT with PdeArAT Complexes and Other ArATs

**Figure 11:**
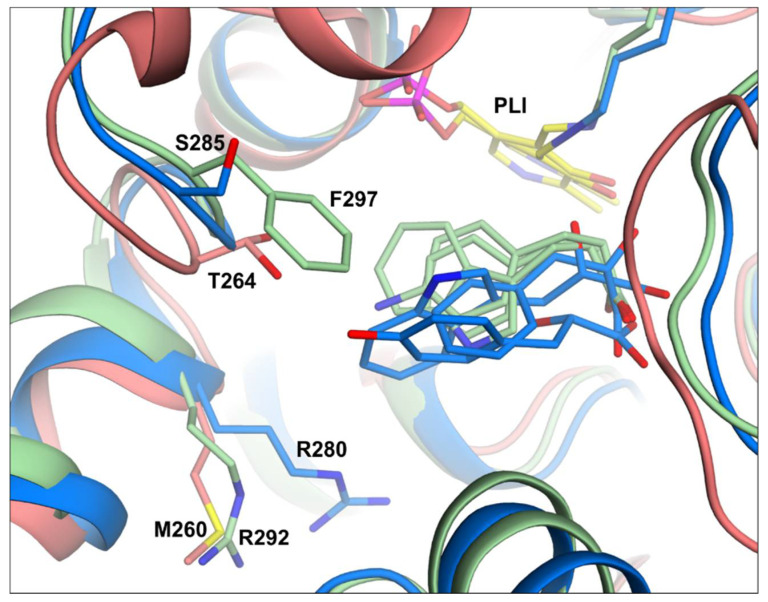
Structural comparison of the active center of bacterial aromatic aminotransferases with ligands: *Psy*ArAT (PDB ids: 6ZUR, 6ZUP, 6ZVG; blue), *Pde*ArAT (PDB ids: 2AY1, 2AY2, 2AY5; green) and *Pho*ArAT (PDB id: 1DJU; salmon). All ligands bound in the active center are shown in the same color as the enzyme peptide chain. In the structure of 1DJU, a ligand was not present.

Structural analysis of *Psy*ArAT in relation to its bacterial homolog from *Paracoccus denitrificans* [20] highlights some similarities. All residues involved in cofactor binding are highly conserved among these two enzymes, as well as amino acids building the smaller compartment of the active pocket being responsible for the binding of substrates by their α-carboxylic group. The most substantial differences is observed in the large chamber of the active site being a place for the ligands’ bulkier parts, responsible for their chemical character. For example, in the place of serine 285 in *Psy*ArAT, there is a phenylalanine 297 in the mesophilic analog *Pde*ArAT (Figure 11). Such a drastic amino acid difference impacts the depth of ligand binding, as aromatic moieties of inhibitors tend not to have enough space.

Broadening the analysis by aromatic aminotransferases from a little more evolutionary distant organisms, thermophilic archaeon (*Pyrococcus horikoshii* (PDB id: 1DJU)) [22] and mesophilic yeast (*Candida albicans* (PDB id: 6HNU)) [21] confirms that many residues in the active pocket are conserved, but they also shows some interesting differences. Residues of the PLP cavity and the smaller compartment of the active pocket were generally the same. Only minor differences, such as the presence of phenylalanine instead of tryptophan in a position of a PLP π-stacking residue, for structures of archaeon and yeast aminotransferases were observed. The most outstanding difference seemed to be a lack of an arginine switch, which was proven to take part in the aromatic aminotransferases’ mechanism of operation. In the case of the *Pho*ArAT structure, the same position was occupied by a methionine residue, having a very different chemical character (Figure 11). In the structure of *Cal*ArAT, the long sidechain of arginine was swapped for a glutamic acid having an opposite charge and a very low chance to operate in the same mechanism.

## 4. Conclusions

In this work, crystal structures of bacterial psychrophilic aromatic amino acid aminotransferases in complexes with four hydroxy-analogs of substrates were presented. Three of the ligands were derivatives of aromatic amino acids: phenylalanine (FOH), tyrosine (YOH) and tryptophan (WOH), and one was of aliphatic aspartic acid (DOH). The partial occupancy of PLP in the structures of FOH and YOH were the result of cofactor loss during the purification process. This is why a crystal of WOH was obtained by co-crystallization with a 20-molar excess of WOH and 5-molar excess of PLP. All ligands, acting as competitive inhibitors, bound in the catalytic pocket allowed for structural analysis of the enzyme active center. Observed structural changes of amino acids side chains in the enzyme catalytic center, as well as the movement of whole domains, mimicked interactions occurring in the active pocket during substrate binding. Although in both monomers of *Psy*ArAT/FOH and *Psy*ArAT/YOH, in the catalytic centers, ligands were bound, we observed differences between conformations of side chains of amino acids in the active pocket in monomers of homodimers. The surface of catalytic pockets in both monomers after binding YOH and FOH differs. For ligands of a similar size and chemical character, changes of the active pocket volume were comparable, but the observed differences between monomers in dimers of the *Psy*ArAT/WOH complex were significantly bigger and the most intriguing. The volume of the pocket in monomer B (D) was twice as big as in monomer A (C) (Figure 8). It was caused by the fact that only one monomer of each dimer bound the ligand (A and C). This different action of catalytic centers in monomers of dimers may indicate that the active centers are somewhat independent while binding bulkier substrates. The small acidic inhibitor (DOH) caused different orientation of the Arg280* and forced closed the conformation of the active center. The results of the structural investigation and activity assay suggest a reverse correlation between activity and gravity of movement of the small domain. The functional dimer of the *Psy*ArAT/DOH complex was created from two symmetry-related subunits. A lack of differences between monomers of this crystallographic dimer allowed for forming of the crystal in a high symmetry space group, with one monomer in the asymmetric unit. The crystallographic experiments performed in this work highlighted the *Psy*ArAT active site’s adaptability towards different-sized substrates.

## Figures and Tables

**Table 1:** Diffraction data collection, processing, and refinement statistics for crystal structures of *Psy*ArAT complexes with substrates hydroxy-analogs.

Data Collection	*Psy*ArAT/DOH	*Psy*ArAT/YOH	*Psy*ArAT/FOH	*Psy*ArAT/WOH
Radiation source	BL.14.2, BESSY, Berlin	BL.14.2, BESSY, Berlin	BL.14.2, BESSY, Berlin	BL.14.1, BESSY, Berlin
Wavelength (Å)	0.9184	0.9184	0.9184	0.9184
Temperature of measurements (K)	100	100	100	100
Detector	Pilatus 3S 2M	Pilatus 3S 2M	Pilatus 3S 2M	Pilatus 3S 2M
Space group	P6_5_22	P2_1_	P2_1_	C2
Cell parameters (Å) a, b, c (Å) α, β, γ (°)	a = b = 90.21 c = 241.75 γ = 120.00	a = 71.67 b = 63.80 c = 82.77 β = 102.66	a = 71.41 b = 60.42 c = 81.96 β = 103.01	a = 177.27 b = 82.29 c = 98.21 β = 106.42
Resolution range (Å)	40.00–1.62	45.62–2.31	50.00–2.49	47.10–2.59
Total no. of reflections	993,205 (57,550)	100,722 (15,260)	79,436 (11,992)	280,530 (42,499)
No. of unique reflections	72,901 (5755)	31,189 (4701)	23,715 (3723)	41,474 (6368)
Completeness (%)	97.02 (78.1)	96.40 (91.0)	98.8 (96.2)	98.3 (94.6)
Redundancy	13.6	3.23	3.35	6.76
I/σ (I)	39.03 (5.73)	9.71 (1.87)	8.29 (0.86)	10.37 (1.16)
Overall B factor from Wilson plot (Å^2^)	21.4	35.5	53.4	64.5
R_merge_ (%)	5.6 (39.0)	9.1 (50.8)	10.3 (135.6)	13.1 (142.9)
R_cryst_/R_free_	12.36/16.37	19.79/24.56	19.89/24.33	19.08/24.74
No. of atoms: Protein/Ion/Ligand/Water	3215/0/24/510	6200/2/93/305	6202/1/71/79	12,228/3/98/163
R.m.s. deviations: Bonds (Å)/Angles (°)	0.02/1.63	0.02/1.64	0.01/1.44	0.01/1.34
Ramachandran plot: Most favored (%)/Allowed (%)	97/3	98/2	96/4	96/4
PDB IDs	6T3V	6ZUR	6ZUP	6ZVG

Values in parentheses correspond to the last resolution shell.

**Table 2:** Parameters showing conformational changes in PsyArAT complexes (for the calculation of the active site volume and area, 1.2 diameter of the probe was used).

	Kink on Long H14 Helix	Shear Movement of C-Terminal	Shear Movement of N-Terminal	Active Site Volume	Active Site Area
PsyArAT/FOH/A	6.0°	2.2 Å	2.7 Å	245 Å3	462 Å2
PsyArAT/FOH/B	4.5°	1.7 Å	3.0 Å	230 Å3	434 Å2
PsyArAT/YOH/A	1.5°	0.5 Å	1.2 Å	315 Å3	518 Å2
PsyArAT/YOH/B	2.0°	1.0 Å	1.8 Å	249 Å3	466 Å2
PsyArAT/WOH/A, C	8.5°	3.3 Å	5.2 Å	186 Å3	406 Å2
PsyArAT/WOH/B, D	0°	0 Å	0 Å	440 Å3	584 Å2
PsyArAT/DOH	9°	3.8 Å	4.5 Å	94 Å3	234 Å2
PsyArAT	reference	reference	reference	412 Å3	598 Å2

## Data Availability

The atomic coordinates of reported crystal structures were deposited in the Protein Data Bank under the accession codes: 6T3V, 6ZUR, 6ZUP and 6ZVG.

## Supplementary Materials

The following are available online at https://www.mdpi.com/article/10.3390/ma14123351/s1, Figure S1: Graphical volume representations of *Psy*ArAT active sites.
