# Supplementary material for: Structural Evidence of Active Site Adaptability towards Different Sized Substrates of Aromatic Amino Acid Aminotransferase from Psychrobacter Sp. B6
Source: Materials (Basel). 2021 Jun 17;14(12):3351. doi: 10.3390/ma14123351 (PMC8235216; doi:10.3390/ma14123351)
Supplement: Supplementary file 1 [file materials-14-03351-s001.zip › materials-1231603-supplementary.pdf]

materials

MDPI

Article

Structural evidence of active site adaptability towards different sized substrates of aromatic amino acid aminotransferase from *Psychrobacter* sp. B6

Anna Bujacz [1,*], Jedrzej Rum [1], Maria Rutkiewicz [2], Agnieszka J. Pietrzyk-Brzezinska [1] and Grzegorz Bujacz [1]

[1] Institute of Molecular and Industrial Biotechnology, Lodz University of Technology, Stefanowskiego 4/10, 90-924 Lodz, Poland; jedrzej.rum@dokt.p.lodz.pl (J.R.); agnieszka.pietrzyk-brzezinska@p.lodz.pl (A.J.P.-B.); grzegorz.bujacz@p.lodz.pl (G.B.)

[2] Macromolecular Structure and Interaction, Max Delbrück Center for Molecular Medicine, Robert-Rössle-Straße 10, 13125 Berlin, Germany; maria.rutkiewicz@mdc-berlin.de

* Correspondence: anna.bujacz@p.lodz.pl

For both subunits A and B of *Psy*ArAT/FOH dimer (Figure S1A,B) volume differences are not big but noticeable (Table 1). In *Psy*ArAT/YOH volume of the active pocket of monomer A (Figure S1C) is bigger than in monomer B (Figure S1D). For the *Psy*ArAT/WOH differences between active site volume are the most visible. Only one monomer of each dimer bound the ligand. The volume of active pocket of monomers A and C with bound inhibitor is much smaller (Figure S1E) in comparison to empty subunits (monomers B and D) (Figure S1F). *Psy*ArAT/DOH crystallized as monomer in asymmetric unit and its active site (Figure S1G) is the smallest, especially comparing to the native *Psy*ArAT (PDB ID: 4RKC) (Figure S1H).

Citation: Bujacz, A.; Rum, J.; Rutkiewicz, M.; Pietrzyk-Brzezinska, A.J.; Bujacz, G. Structural Evidence of Active Site Adaptability Towards Different Sized Substrates of Aromatic Amino Acid Aminotransferase from *Psychrobacter* sp. B6. *Materials* **2021**, *14*, 3351. https://doi.org/10.3390/ma14123351

Academic Editor(s): Shouke Yan

Received: 06 May 2021
Accepted: 09 June 2021
Published: 17 June 2021

Publisher's Note: MDPI stays neutral with regard to jurisdictional claims in published maps and institutional affiliations.

Copyright: © 2021 by the authors. Licensee MDPI, Basel, Switzerland. This article is an open access article distributed under the terms and conditions of the Creative Commons Attribution (CC BY) license (http://creativecommons.org/licenses/by/4.0/).

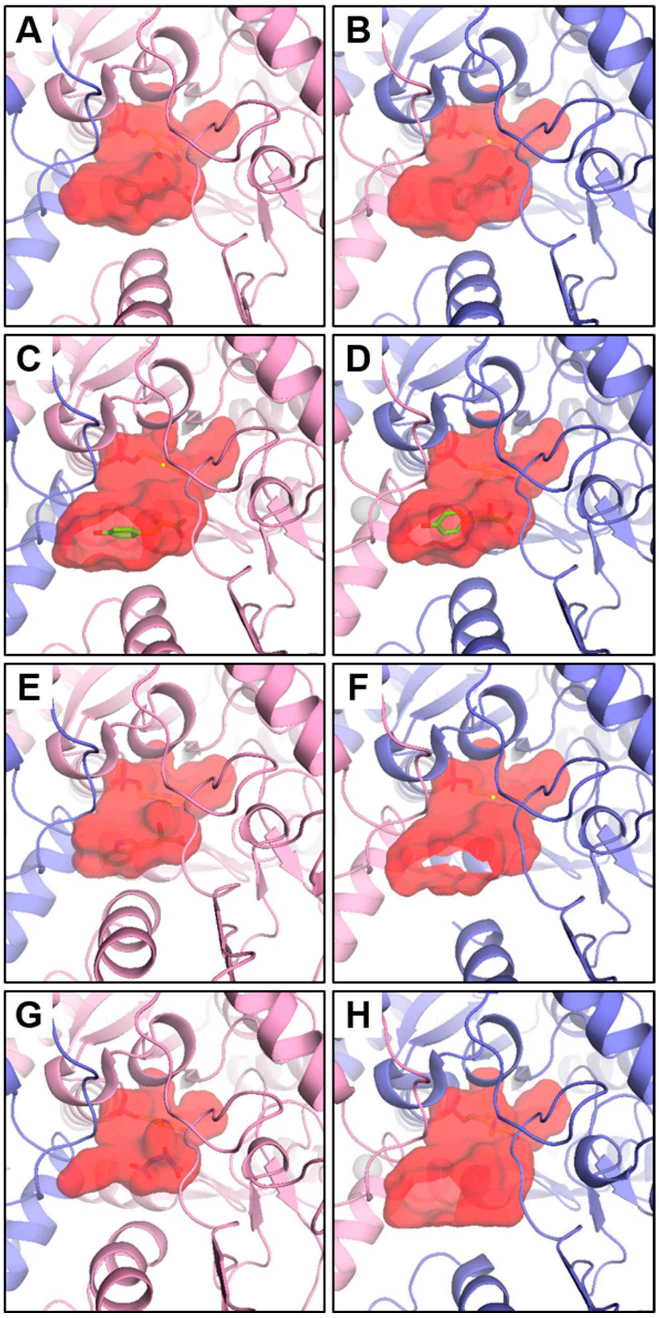

Figure S1. Graphical volume representations of *Psy*ArAT active sites. (**A**, **B**) *Psy*ArAT/FOH monomer A and B, respectively; (**C**, **D**) *Psy*ArAT/YOH monomer A and B, respectively; (**E**, F) *Psy*ArAT/WOH - monomer A and B, respectively; (**G**) *Psy*ArAT/DOH monomer; (**H**) *Psy*ArAT (PDB ID: 4RKC) monomer B.
